# Supplementary figures and images for: Glioblastoma signature in the DNA of blood-derived cells
Source: PLoS One. 2021 Sep 8;16(9):e0256831. doi: 10.1371/journal.pone.0256831 (PMC8425531; doi:10.1371/journal.pone.0256831)

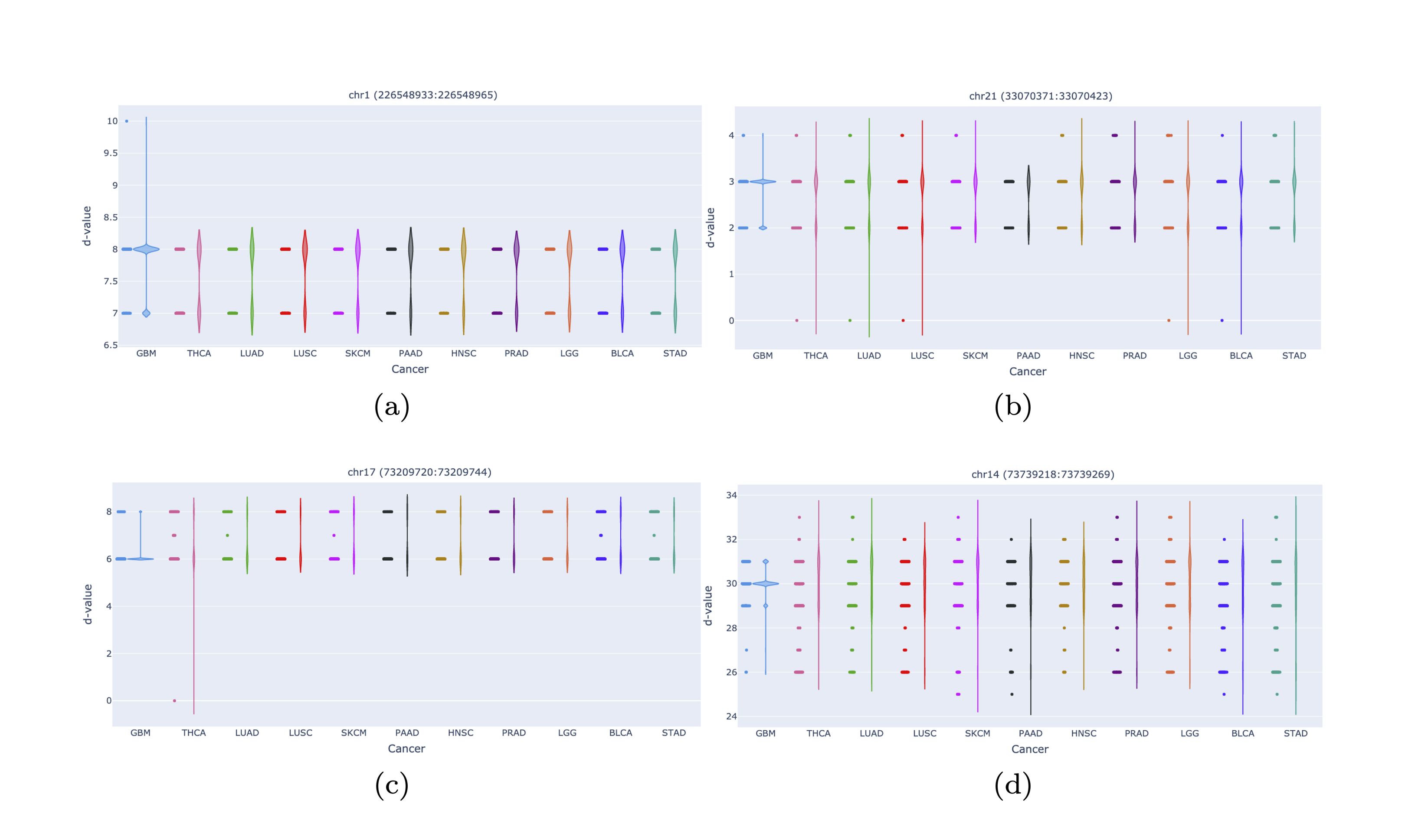

Supplement: S1 Fig — Violin plots representing the distribution of m or d in tandem repeat areas in the DNA derived from blood cell for patients with different cancer types. These tandem repeat areas show distinctive distribution of m or d values. (TIF) [file pone.0256831.s001.tif]

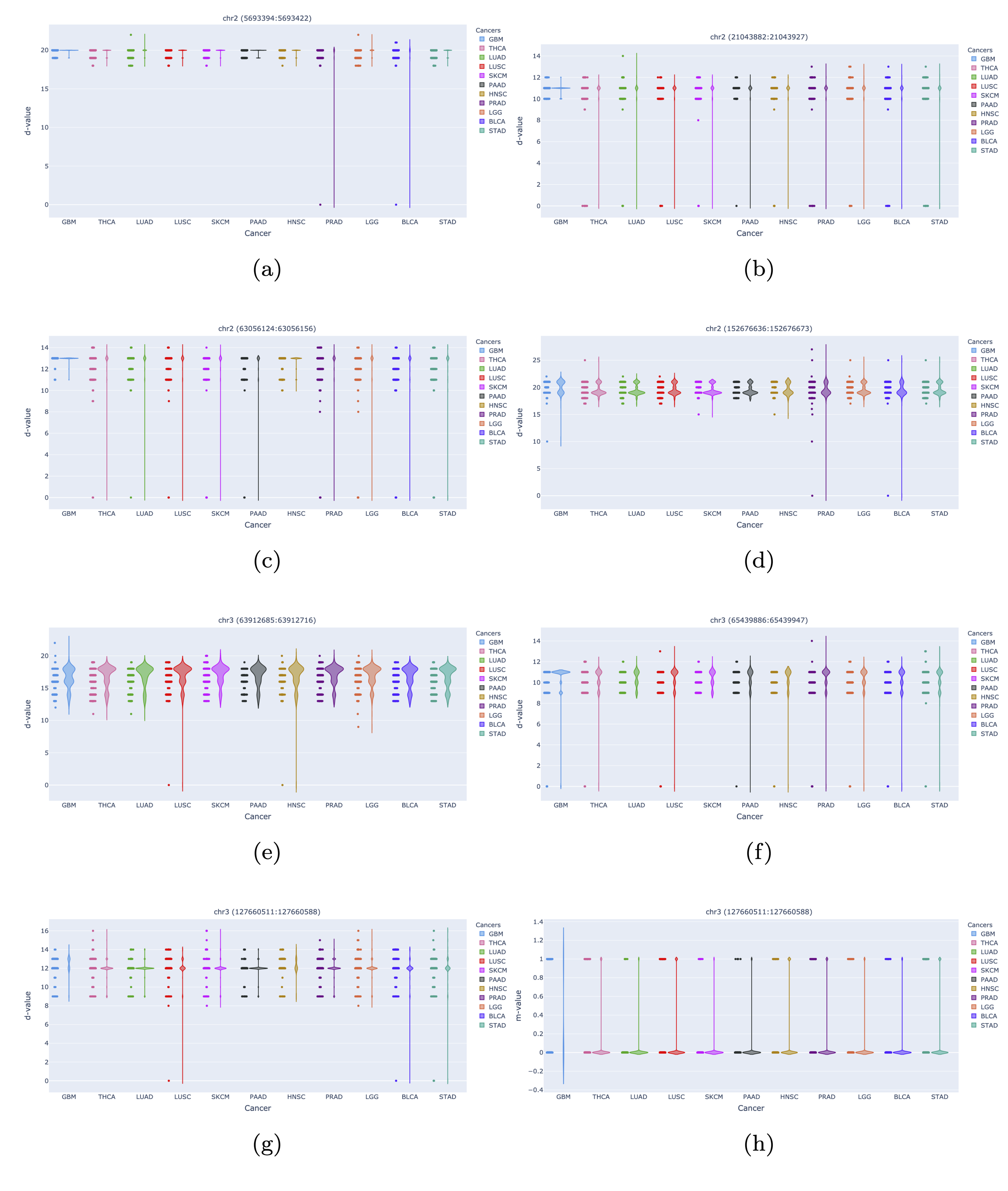

Supplement: S2 Fig — Violin plots representing the distribution of m or d in tandem repeat areas in the DNA derived from blood cell for patients with different cancer types. These tandem repeat areas show distinctive distribution of m or d values. (TIF) [file pone.0256831.s002.tif]

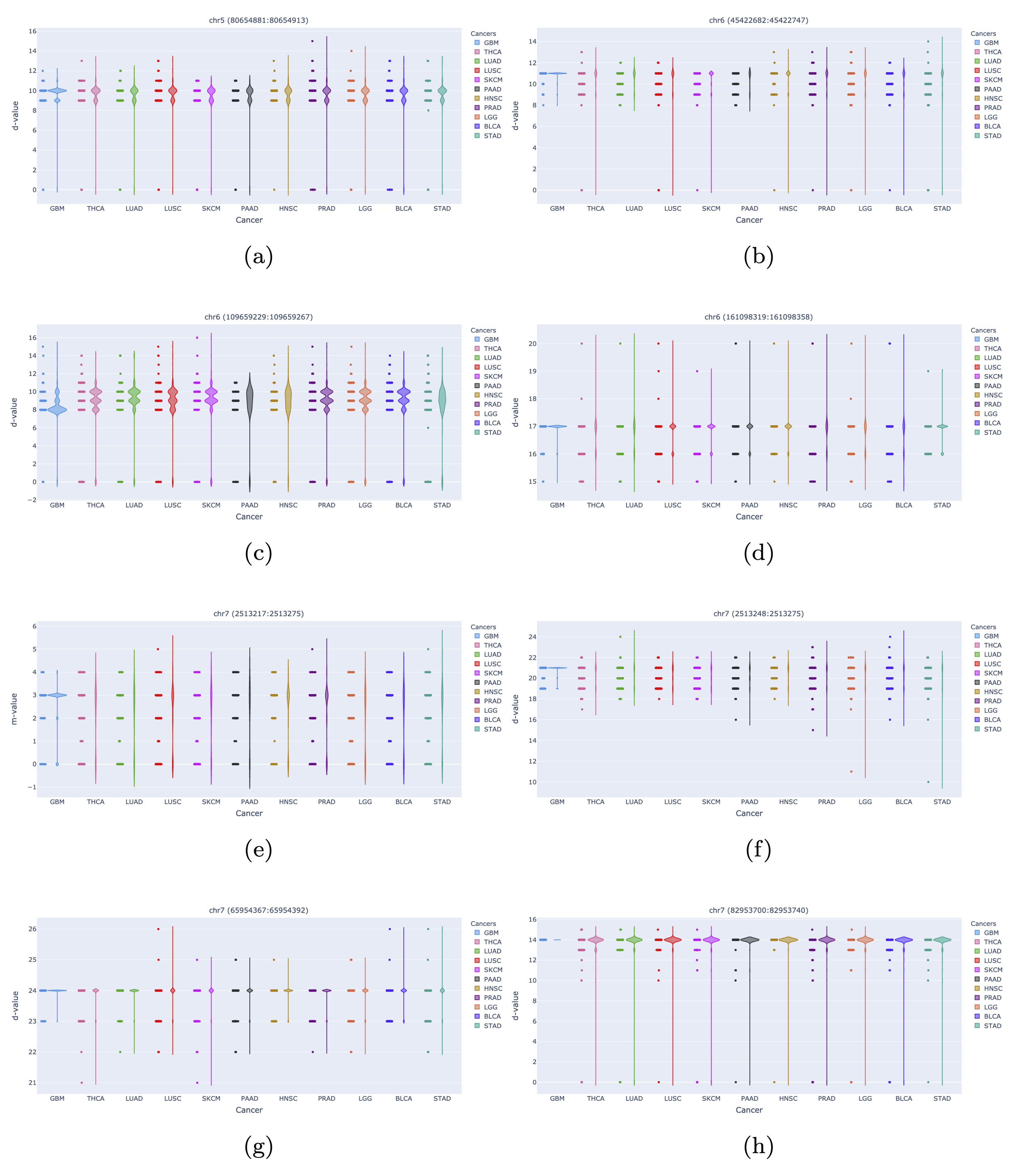

Supplement: S3 Fig — Violin plots representing the distribution of m or d in tandem repeat areas in the DNA derived from blood cell for patients with different cancer types. These tandem repeat areas show distinctive distribution of m or d values. (TIF) [file pone.0256831.s003.tif]

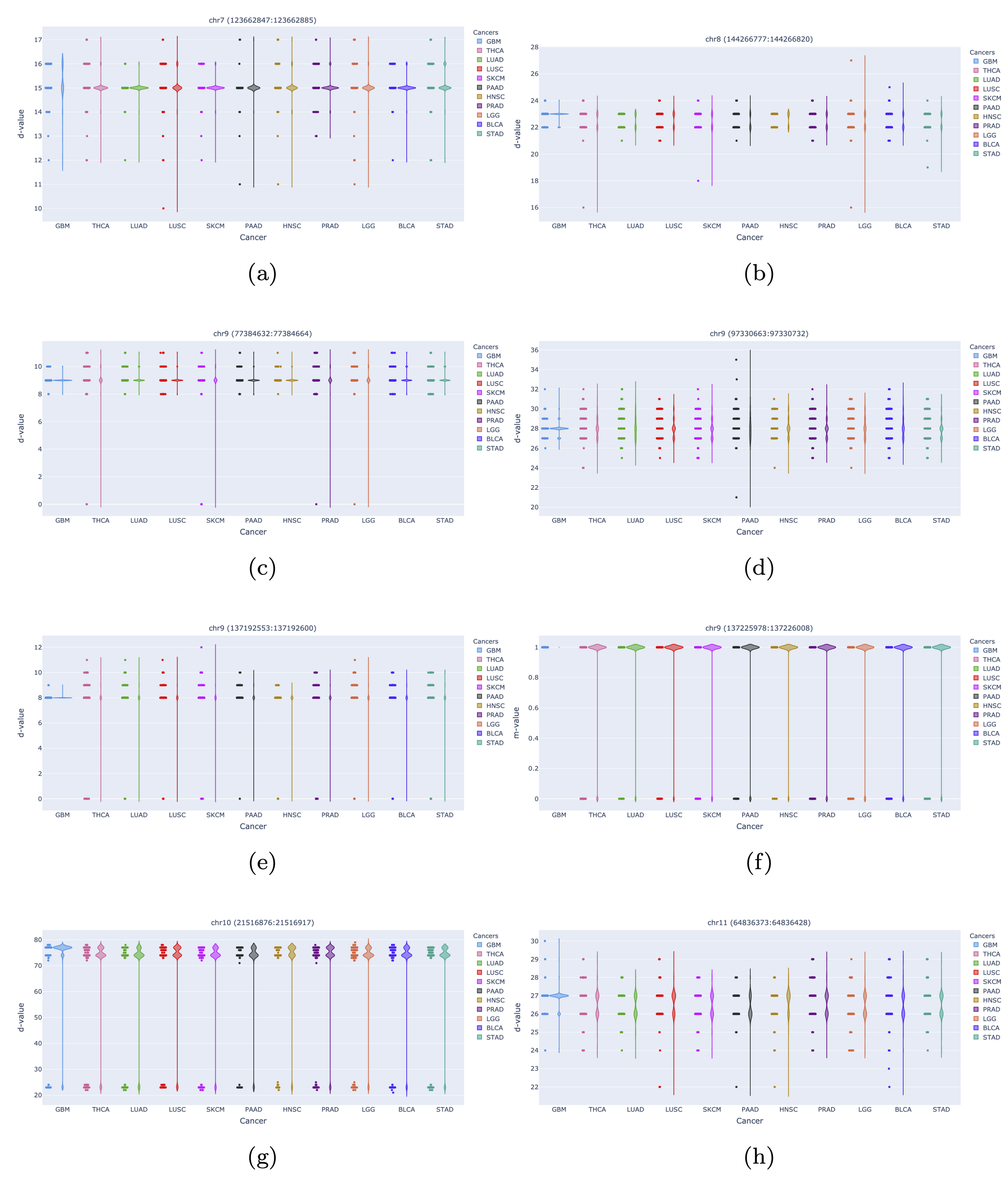

Supplement: S4 Fig — Violin plots representing the distribution of m or d in tandem repeat areas in the DNA derived from blood cell for patients with different cancer types. These tandem repeat areas show distinctive distribution of m or d values. (TIF) [file pone.0256831.s004.tif]

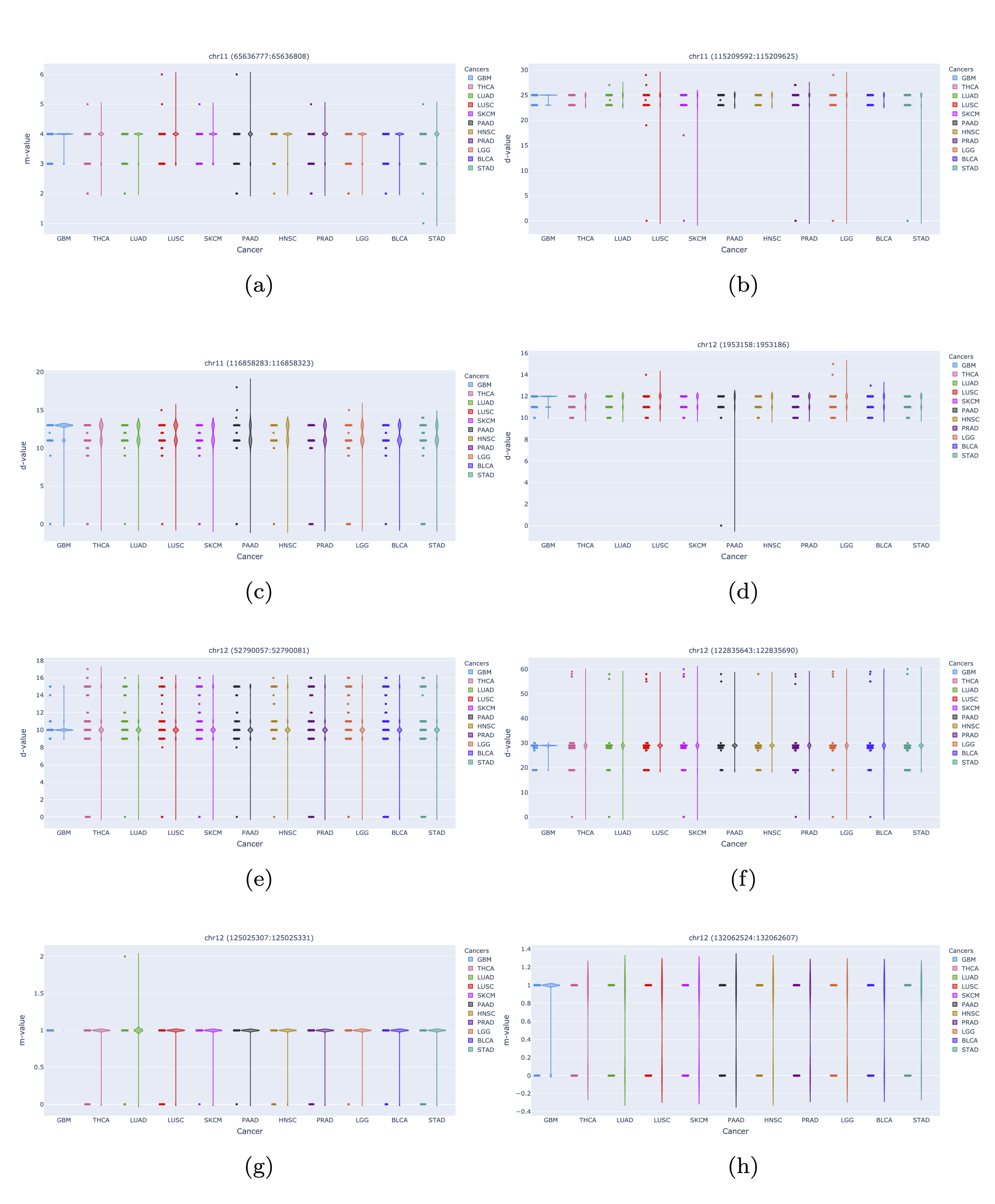

Supplement: S5 Fig — Violin plots representing the distribution of m or d in tandem repeat areas in the DNA derived from blood cell for patients with different cancer types. These tandem repeat areas show distinctive distribution of m or d values. (TIF) [file pone.0256831.s005.tif]

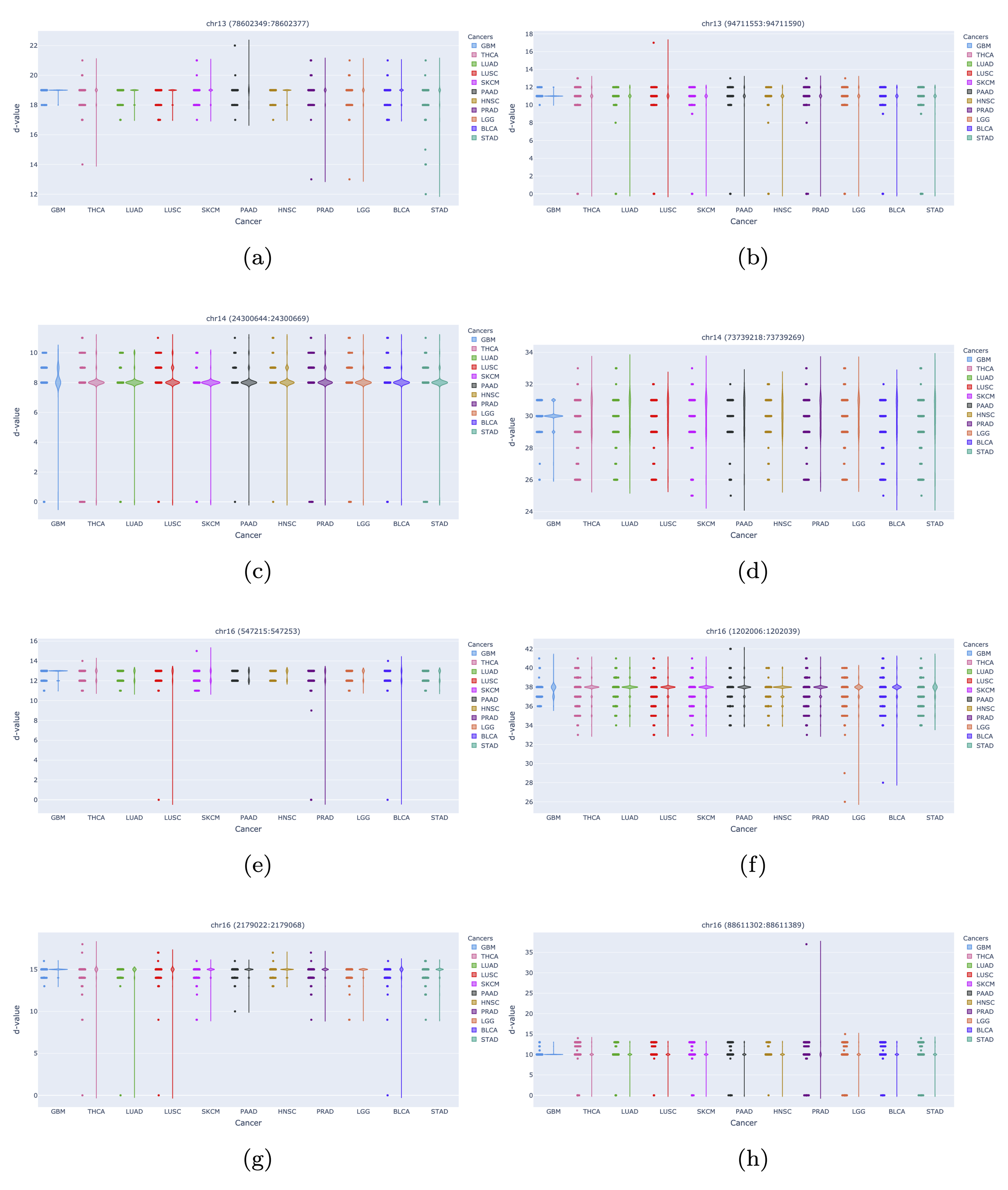

Supplement: S6 Fig — Violin plots representing the distribution of m or d in tandem repeat areas in the DNA derived from blood cell for patients with different cancer types. These tandem repeat areas show distinctive distribution of m or d values. (TIF) [file pone.0256831.s006.tif]

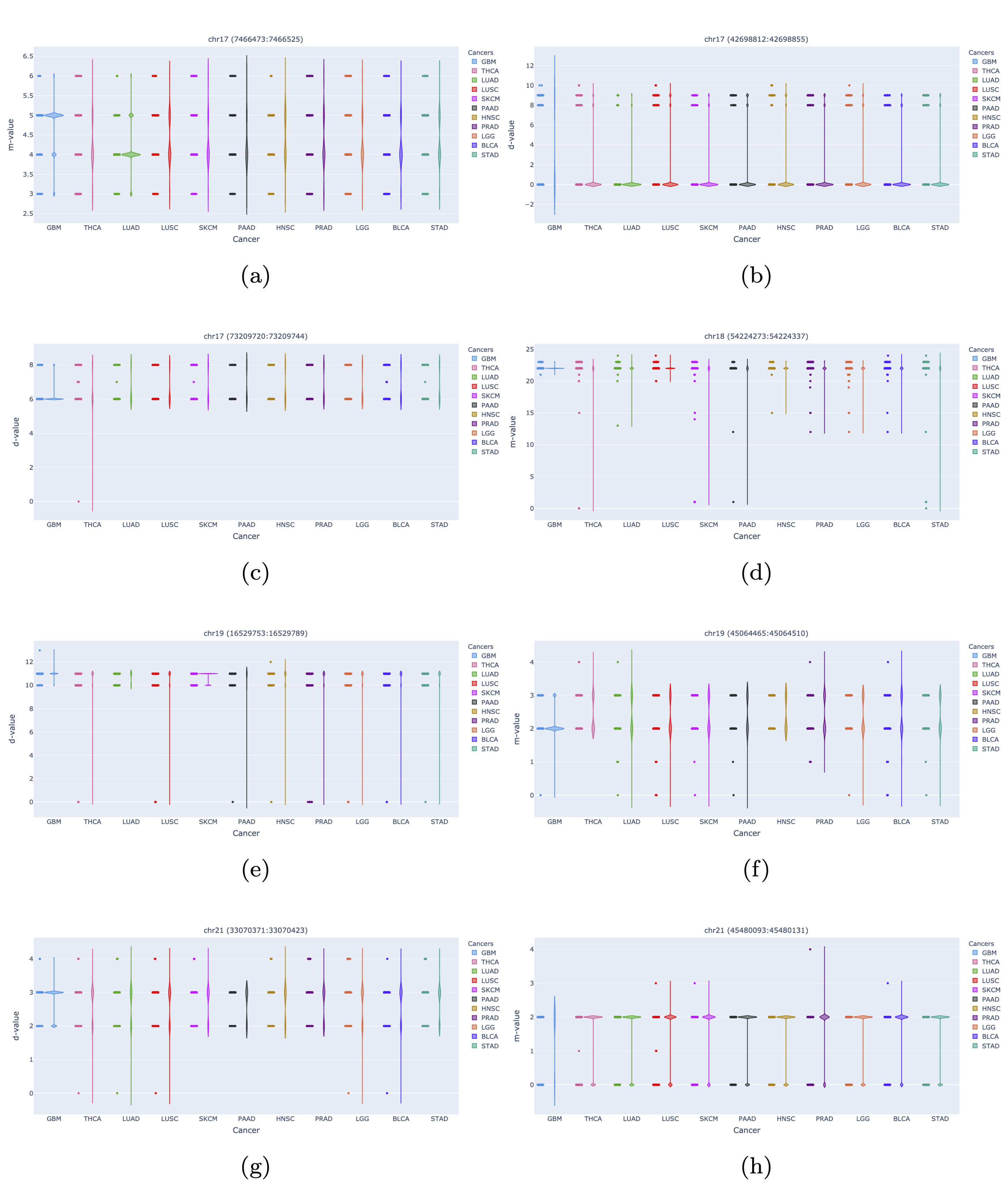

Supplement: S7 Fig — Violin plots representing the distribution of m or d in tandem repeat areas in the DNA derived from blood cell for patients with different cancer types. These tandem repeat areas show distinctive distribution of m or d values. (TIF) [file pone.0256831.s007.tif]

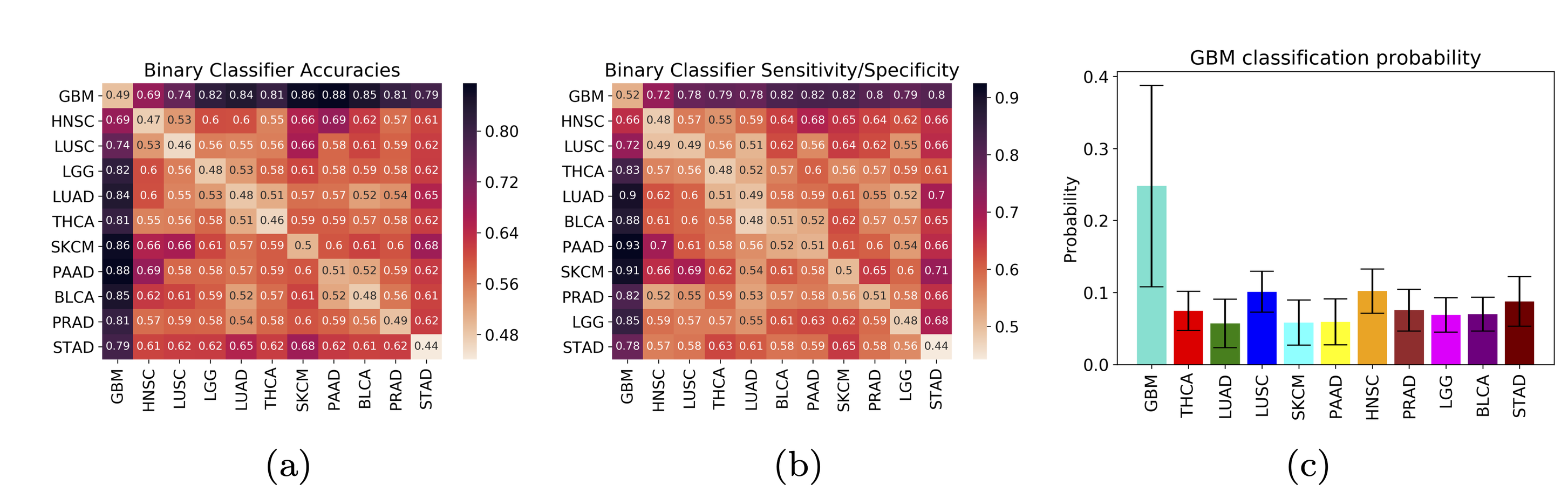

Supplement: S8 Fig — Accuracy, Sensitivity and Specificity for Pairwise and Multi Classifiers among different cancer types: Seriation diagram for the pairwise classifiers showing the presence of distinguishing signal between GBM and other cancer types (darker cells) using (a) Mean Validation accuracy and (b) Sensitivity and Specificity. Mean validation accuracy ranges from 69% to 88% when GBM is compared against different cancers in (a). Mean sensitivity ranges from 72% to 82% when GBM is compared against other cancers in (b). Mean specificity ranges from 66% to 93% when GBM is compared against different cancers in (b). A multiclassifier built to compare mutation profiles of GBM patients with other cancer types, here we show that the mutliclassifier is successful in classifying GBM patients using the Multiclassification probability profile in (c). (TIF) [file pone.0256831.s008.tif]
