# Supplementary material for: Glioblastoma signature in the DNA of blood-derived cells
Source: PLoS One. 2021 Sep 8;16(9):e0256831. doi: 10.1371/journal.pone.0256831 (PMC8425531; doi:10.1371/journal.pone.0256831)
Supplement: Attachment — Submitted filename: PLOS_rebuttal.docx [file pone.0256831.s020.docx]

We thank the reviewers and the editorial board for a careful review of our manuscript. Below, we address the concerns raised:

1. Data availability restrictions: The data is owned by The Cancer Genome Atlas consortium. Since, the DNA data involves human subjects, the data has controlled access and can be accessed by applying using dbGap (https://dbgap.ncbi.nlm.nih.gov/aa/wga.cgi?page=login) under accession code phs000178v1.p1. When we mentioned that the data is available upon request, we meant that the data can be requested using dbGap. Our access to the data also required approval by the Caltech Institutional Review Board. The information about TCGA sample file name and file id used in our analysis is provided in the supplementary CSV files for different cancer types. We have modified the cover letter with these details.
2. Reviewer 1

- We have removed the use of term healthy from the paper and instead use blood-derived DNA. We have also changed the title of our paper to **“*Glioblastoma Signature in the DNA of Blood-Derived Cells’’.*** However, we also want to point out that we carried out the same analysis with DNA derived from normal tissue and obtained similar results. We didn’t include that analysis in the main manuscript since we wanted to point out the existence of indicative cancer signals in blood-derived DNA. Further, our hypothesis is the blood-derived DNA of healthy people may have indicative signals for their future cancer risk. However, this hypothesis can only be validated if access to blood-derived DNA of cancer patients can be obtained when they were healthy.
- We agree with the reviewer that we are proposing a new computational technique to analyze DNA, however in this paper the focus is on the implication of this computational technique on inferring the presence of signal for Glioblastoma in blood-derived DNA.

3. Reviewer 2

- We would have liked to work with whole genome samples, however TCGA only has Whole Exome samples (WXS). Therefore, we only worked with exomic regions. The method is applicable to whole genome samples and we believe that if one can obtain whole genome samples, the nature of inferred signal for GBM and other cancers might be stronger than what is being reported in the current manuscript.
- “healthy people DNA”: The reviewer is correct in making this point. There are two reasons, why we didn’t include healthy people in our analysis:

(i) The WXS data on TCGA is high quality and has a coverage of 30-40X. We couldn’t find a large DNA dataset of healthy people of the aforementioned quality. We looked at 1000 Genome data, however the coverage of that data is very low compared to TCGA data.
(ii) The second reason is a bit speculative and philosophical. We hypothesize that the blood-derived DNA of a currently healthy individual may have signals for cancer of some type(s) that they can get in the future. If this hypothesis is correct, it may mean, that the DNA of a currently healthy individual may also have signals for cancer(s) they may get in the future. Hence for the purpose of the analysis reported in the manuscript, the individual might fall in a category of some cancer type.

- We have changed bold font to color red in the new version.

We have also added some text (highlighted in blue) in the Discussion section summarizing some of the points raised by the reviewers. All the changes made in the manuscript are highlighted in blue.

Thanking you,

Yours sincerely,

Siddharth Jain, Bijan Mazaheri, Netanel Raviv, Jehoshua Bruck
